# A Reminder App to Optimize Bladder Filling During Radiotherapy for Patients With Prostate Cancer (REFILL-PAC): Protocol for a Prospective Trial

**DOI:** 10.2196/68179

**Published:** 2025-04-08

**Authors:** Dirk Rades, Jan-Dirk Küter, Michael von Staden, Ahmed Al-Salool, Stefan Janssen, Carmen Timke, Marciana Nona Duma, Tobias Bartscht, Christine Vestergård Madsen, Charlotte Kristiansen, Florian Cremers

**Affiliations:** 1 Department of Radiation Oncology University Medical Center Schleswig-Holstein, Campus Lübeck Lubeck Germany; 2 Department of Radiation Oncology University of Lübeck Lubeck Germany; 3 Medical Practice for Radiotherapy and Radiation Oncology Hannover Germany; 4 Department of Radiotherapy Malteser Hospital St Franziskus Flensburg Germany; 5 Department of Radiotherapy Helios Hospital Schwerin Schwerin Germany; 6 Department of Hematology, Oncology and Stem Cell Transplantation Helios Hospital Schwerin Schwerin Germany; 7 Department of Oncology Vejle Hospital University Hospital of Southern Denmark Vejle Denmark

**Keywords:** prostate cancer, external beam radiation therapy, radiation toxicity, bladder filling, mobile app

## Abstract

**Background:**

Many patients with nonmetastatic prostate cancer receive radiotherapy, which may be associated with acute cystitis, particularly if the volume of the urinary bladder is small. Three studies showed bladder volumes <200 ml or <180 ml to be associated with increased urinary toxicity. Therefore, it is important to maintain bladder volumes greater than 200 ml during as many radiation fractions as possible. Several studies investigated drinking protocols, where patients were asked to drink a certain amount of water prior to radiotherapy sessions. This may require considerable discipline from the patients, who are predominantly older adults. Adherence to a drinking protocol may be facilitated by a mobile app that reminds patients to drink water prior to each radiation session. This study investigates the effect of such an app on bladder filling status in patients with prostate cancer undergoing external beam radiotherapy (EBRT) alone.

**Objective:**

The primary goal of this study is to evaluate the impact of an app that reminds patients irradiated for prostate cancer to drink 300 ml of water prior to each radiotherapy session on the number of fractions with bladder volumes <200 ml during the radiotherapy course.

**Methods:**

This ongoing phase 2 aims to recruit 28 patients treated with EBRT alone for nonmetastatic prostate cancer. Radiotherapy will be administered using normo-fractionation, with doses ranging from 70 to 80 Gy in 35 to 40 fractions of 2 Gy, preferably with volumetric-modulated arc therapy (VMAT). Treatment volumes include the prostate with or without the seminal vesicles.

**Results:**

Recruitment for this trial will start in March 2025 and is planned to be completed in October 2026. The study is scheduled to conclude in December 2026.

**Conclusions:**

This trial is the first to evaluate the impact of a reminder app on the number of radiotherapy fractions with bladder volumes <200 ml in patients undergoing irradiation for localized prostate cancer.

**Trial Registration:**

Clinicaltrials.gov NCT06653751; https://clinicaltrials.gov/show/NCT06653751

**International Registered Report Identifier (IRRID):**

PRR1-10.2196/68179

## Introduction

Prostate cancer is one of the most common solid malignancies worldwide [1]. Most patients with nonmetastatic disease receive either prostatectomy or radiotherapy. Radiotherapy is often performed with normo-fractionated (5 × 2 Gy per week) external beam radiotherapy (EBRT) alone or EBRT plus a high-dose-rate brachytherapy boost [2]. For normo-fractionated EBRT alone, the recommended total dose is between 74 and 80 Gy, corresponding to 37 to 40 fractions of 2 Gy [2]. Prostate cancer radiotherapy may be associated with significant acute urinary toxicity such as cystitis, particularly if the volume of the urinary bladder is small. A total of 3 studies showed that bladder volumes <200 ml or <180 ml, respectively, were associated with increased acute or late urinary toxicity [3-5]. In a retrospective study by Pisani et al [3], which included 280 patients treated with EBRT for prostate cancer, bladder filling (volumes <200 ml vs ≥200 ml) was an independent predictor of grade ≥2 acute urinary toxicity. A prospective study by Pinkawa et al [4] assessed the quality of life in 80 patients irradiated for prostate cancer at different time points. At follow-up between 6 and 10 weeks after radiotherapy, pain with urination was reported less frequently by patients with an initial bladder volume of ≥180 ml compared to <180 ml. In another prospective study from Germany, 193 patients received training via a biofeedback mechanism to achieve a bladder volume between 200 and 300 ml at each radiation session [5]. The results showed that reproducible bladder volumes >180 ml were associated with a significant decrease in grade ≥2 acute urinary toxicity. In another study, a planned bladder volume >200 ml and daily filling between 82% and 113% were associated with reduced intrafraction motion of the prostate [6]. Moreover, in the study by Smith et al [7], optimal bladder dose constraints were missed more frequently if the bladder volumes were <200 ml. Therefore, it is important to maintain bladder volumes >200 ml at as many radiation fractions as possible.

In a recent study by our group that investigated the bladder volumes at each of the first 35 radiation fractions in 72 patients receiving EBRT alone, the mean and median numbers of radiation fractions with bladder volumes <200 ml were 17.8 (SD 12) and 16.5 (IQR 7.5-29.5) fractions, respectively [8]. On the other hand, in a subgroup of 37 patients with a bladder volume of at least 200 ml before the start of the radiotherapy course, the mean and median numbers of radiation fraction with bladder volumes <200 ml were only 9.4 (SD 8.3) and 8 (IQR 2-16), respectively. The mean and median were significantly higher in the subgroup of 35 patients with bladder volumes <200 ml, at 26.7 (SD SD 8.5) and 30 (IQR 22-34), respectively. Therefore, there is a medical need for improvement, especially in the latter subgroup.

Several studies have investigated the role of drinking protocols [5-7,9-17]. Patients were asked to drink a certain amount of water prior to computed tomography (CT) simulation and radiotherapy sessions. In these studies, the amount of water ranged between 200 and 600 ml, and the time interval until the CT simulation or radiation session was between 30 and 60 minutes [5-7,9-17]. However, drinking a certain amount of water at a specific point in time may require considerable discipline from patients, who are predominantly older adults. These considerations led to the idea of developing a mobile app that reminds patients to drink a certain amount of water prior to each radiation session. The idea of testing a reminder app in this context was based on our experience with such an app during our previous Interreg project NorDigHealth [18,19].

This study investigates the number of radiation fractions with bladder volumes <200 ml in a prospective cohort of patients using a reminder app. Additionally, it evaluates whether the use of the reminder app significantly reduces the proportion of radiation fractions with bladder volumes <200 ml compared to a historical control group without app support. After several ethical discussions, we decided to compare the cohort of the single-arm phase 2 trial to an appropriate historical control group after careful matching rather than performing a randomized phase 3 trial. Since the responsible individuals at the contributing centers were confident that the reminder app would improve bladder filling in the phase 2 cohort, they deemed it unethical to withhold the app from approximately 50% of the patients participating in a randomized phase 3 trial. The main goal of this prospective phase 2 study is to evaluate the impact of a reminder app on the number of fractions with bladder volumes <200 ml during radiotherapy in patients irradiated for prostate cancer. The app reminds the patients to drink 300 ml of water 45 minutes prior to each radiotherapy session. The primary end point is the number of radiation fractions with bladder volumes <200 ml after 35 fractions of radiotherapy. Additionally, the following end points will be evaluated: (1) the number of radiation fractions with bladder volumes <200 ml at the end of radiotherapy, (2) patient satisfaction with the reminder app, and (3) the impact of the reminder app on the use of health technology.

## Methods

### Trial Design and Duration

This is a single-arm prospective study performed in 1 university hospital, 2 academic teaching hospitals, and 1 private practice. It will investigate the effect of a reminder app on the number of radiation fractions with bladder volumes <200 ml during a course of radiotherapy for the treatment of prostate cancer compared to a historical control group [8]. The control group is considered appropriate for comparison with this study cohort, as these patients were treated very recently (in 2022 or 2023) with external beam radiotherapy alone in 3 of the 5 centers participating in this study. The control group underwent a cone beam computed tomography (CBCT) session prior to each radiation fraction, enabling bladder volume assessment with the same level of precision as in this study. To ensure comparability of the total number of fractions administered in the prospective study with those in the historical control group, the primary end point is restricted to the first 35 fractions administered. The recruitment of all 28 patients is planned to be completed within 20 months. The follow-up period will end directly after the radiotherapy course, which is scheduled to take 7 to 8 weeks. This equals a total running time of 22 months for the study. In accordance with the previous study assessing the number of radiation fractions with bladder volumes <200 ml during a course of radiotherapy for treating prostate cancer, the following characteristics will be recorded to allow adequate comparison with the historical control group: (1) bladder volume at CT simulation, (2) BMI, (3) age, (4) prostate volume prior to radiotherapy, (5) Karnofsky performance score, (6) risk group of prostate cancer, and (7) antihormonal therapy [8]. All patients in the phase 2 cohort and the historical control group must have a bladder volume <200 ml at CT simulation.

### Patient Selection

This trial will be performed in patients with prostate cancer receiving definitive normo-fractionated radiotherapy. Patients will be adequately informed about their diagnosis and the nature, significance, and scope of the trial. Patients will only be included after completing the pretherapy evaluation, meeting all inclusion criteria, and not meeting any exclusion criteria (Textbox 1).

**Inclusion and exclusion criteria.**

**Inclusion criteria:**

- Histologically proven prostate cancer
- Indication for definitive normo-fractionated radiotherapy
- Possession of and ability to use a smartphone
- Bladder volume at computed tomography (CT) simulation <200 ml
- Age ≥18 years
- Written informed consent
- Capacity of the patient to consent

**Exclusion criteria:**

- Radiotherapy of pelvic lymph nodes
- Expected noncompliance

### Patient Interventions

For all patients, radiotherapy will be administered using normo-fractionation with 70 to 80 Gy in 35 to 40 fractions of 2 Gy, given 5 days per week (overall treatment time 7-8 weeks), preferably with volumetric-modulated arc therapy (VMAT) [2]. Treatment volumes include the prostate with or without the seminal vesicles.

Radiotherapy for prostate cancer may be associated with acute side effects, including dermatitis, cystitis, proctitis, diarrhea, and fatigue. If grade 3 toxicity occurs according to the Common Terminology Criteria for Adverse Events (CTCAE) version 5, radiotherapy may be delayed for a maximum of 7 days without consequences [20]. If it is delayed for more than 7 days, participation in the study will be terminated, and the coordinating investigator must be informed.

Patients may receive concurrent systemic agents as part of their anticancer treatment, regardless of their participation in this trial [2]. These agents will be indicated and prescribed by treating medical oncologists or urologists outside this trial.

The following parameters will be recorded prior to the start of radiotherapy: medical history including micturition disorders, medication including anticancer treatment, physical examination, age, body height and weight, Karnofsky performance score, bladder volume and prostate volume at CT simulation, tumor stage, histology, Gleason score, prostate-specific antigen, risk group, planned radiation dose and dose per fraction, number of fractions, radiation boost, treatment volume, radiation technique, experience with smartphones, and need for support regarding the use of the reminder app. Throughout the trial, bladder volumes will be assessed by staff members prior to the radiotherapy course (based on CT simulation) and before each radiation fraction using CBCT. Adverse events will be assessed on an ongoing basis according to CTCAE version 5 [20]. At the end of the radiotherapy course, patients will be asked to complete a questionnaire assessing their satisfaction with the reminder app and the impact of its use on their attitude toward health technology.

### Safety Management

An adverse event is any event experienced by a patient or participant in a clinical trial that does not necessarily have a causal relationship with the treatment. It can include any adverse or inadvertent occurrence (including notable laboratory findings), symptom, or illness that occurs during the treatment period, regardless of whether there is a causal relationship. Existing illnesses that deteriorate during the trial should also be reported as adverse events. Events covered by the documentation for concomitant diseases and radiation-related acute toxicities of Grade ≤2 do not have to be additionally documented as adverse events. Serious adverse events are those that fulfill one of the following criteria at any dose level: lethal, life-threatening, requiring hospitalization or extent of a hospital stay, permanent or significant disability, birth defects or malformations, any medically significant event, or any event necessitating surgery to prevent one of the aforementioned concomitant illnesses. Hospitalization should be defined as necessary for treating the adverse event. Hospital stays that are part of the treatment outlined in the protocol or due to a planned, elective operation are not classified as serious adverse events. Likewise, an elective hospitalization to facilitate the trial process does not count as a serious adverse event.

### Sample Size Calculation

The primary goal of this study is to assess the impact of an app that reminds patients irradiated for prostate cancer to drink water before each radiotherapy session on the number of fractions with bladder volumes <200 ml during the radiotherapy course. This study also aims to demonstrate that this number is lower than without using an app (historical control group). To allow for a skewed distribution of the primary end point, the Wilcoxon-Mann-Whitney test will be applied for confirmatory statistical analysis. Sample size calculation is based on the article by Noether [21]. In the external historical control group consisting of 35 patients, the mean number of radiation fractions with bladder volumes less than 200 ml was 26.7 (SD 8.5), and the median number was 30 (IQR 22-34). A decrease in this mean value by roughly 30% (to 18.7 fractions) is considered clinically relevant. For illustrative purposes, translating this decrease into a nonparametric effect size framework (assuming normal distribution) leads to a probability of roughly 0.25 that the number of fractions <200 ml with the reminder app will be larger than without the app. Based on this effect size, a sample size of 25 patients in the prospective trial is required for comparison with the historical control group to ensure 90% power to reach statistical significance with a 2-sided Wilcoxon-Mann-Whitney U test and a 5% significance level. Assuming that roughly 10% of the enrolled patients will not be eligible for the primary analysis as they received less than 35 fractions, a total of 28 patients should be enrolled.

### Statistical Analyses

All data recorded in the case report forms describing the study population, efficacy, safety, and quality of life will be analyzed descriptively. Categorical data will be presented in contingency tables with frequencies and percentages and 95% CIs. Continuous data will be summarized with at least the following: frequency (n), median, quartiles, mean, SD (standard error), minimum, and maximum. The number of participants with protocol deviations during the study and listings describing the deviations will be provided. Chi-square tests will be used to compare percentages in a 2 × 2 contingency table, replaced by the Fisher exact test if the expected frequency in at least 1 cell of the associated table is less than 5. Stratified 2 × 2 contingency tables will be analyzed using Cochran-Mantel-Haenszel tests. Logistic regression models serve as multivariable methods for binary end point data. A comparison of ordinal variables between treatment arms will be performed using the asymptotic Wilcoxon-Mann-Whitney test, replaced by its exact version in case of ordinal categories with a small number of categories and/or sparse data within categories. Any shift in the location of quantitative variables between study groups will be performed using the Wilcoxon-Mann-Whitney tests. All patients who start the radiotherapy and provide data on the primary end point will be analyzed (full analysis set). The data analysis will be performed according to the statistical analysis plan, which will be finalized prior to database lock and any statistical analysis.

The primary study end point is defined as the number of radiation fractions with bladder volumes less than 200 ml after 35 fractions of radiotherapy. Descriptive measures of location and dispersion will be used to describe the results of the prospective study. The impact of patient characteristics on the primary end point will be assessed by Wilcoxon 2-sample tests. These characteristics include age (<75 vs ≥75 years), Karnofsky performance score (70-80 vs 90-100), BMI (<30 vs ≥30=obesity), prostate volume prior to radiotherapy (<60 vs ≥60 mL), risk group of prostate cancer (low to intermediate vs high), and antihormonal therapy prior to and/or during the course of radiotherapy (no vs yes). For confirmatory analysis, the prospective study will be compared with the historical control group through a 2-sided Wilcoxon-Mann-Whitney 2-sample test and a significance level of 5%. A high degree of comparability is expected between the prospective trial cohort and the retrospective patient data set. However, potential heterogeneity among the study populations will be identified by comparing patient characteristics with Wilcoxon-Mann-Whitney tests. Homogeneity will be assumed if all resulting *P* values are above 20%. Any factor indicating a tendency toward heterogeneity (ie, *P*<.20) will be included in a multivariable count data Poisson regression model with the number of radiation fractions with bladder volumes <200 ml as a dependent variable and the respective factors and binary factor (prospective study vs historical control) as the independent variables. If there is evidence of overdispersion, the Poisson model will be replaced by a negative binomial model.

Additionally, secondary end points will be subjected to statistical analysis. Since no comparison with historical data is possible, these analyses will focus on descriptive statistical analysis only. Patient satisfaction results will be used to decide whether the app needs modifications. In the case of a dissatisfaction rate >20%, app modifications will be made. If the app has a dissatisfaction rate >40%, it will be considered not useful.

### Ethical Considerations

The examinations to be carried out as part of this trial are all considered standard procedures. There are no additional laboratory investigations or X-rays to be done, or any other examinations that could be potentially burdensome for the patient. The trial protocol was approved by the ethics committee of the University of Lübeck, Germany (file 2024-519), and the trial has been registered at Clinicaltrials.gov (NCT06653751). Prior to inclusion in the trial, each patient will be fully informed about its contents and procedures. If the patient has received the necessary information and the investigator is sure that the patient has understood this information, they will be asked to provide their consent via signature. The patient will receive a copy of their information and the signed informed consent forms. The investigator will also inform the patient that they have the right to withdraw consent to participate in the trial at any time and without providing any reasons. Patients will be informed that the data collected as part of the trial will be documented anonymously and then forwarded for scientific evaluation.

The trial will be conducted in accordance with the principles laid out in the Declaration of Helsinki. Data will be collected in accordance with the regulations set out in the Data Protection Act. All findings from the clinical trial will be stored on electronic data storage devices and treated with utmost confidentiality. Organizational measures have been taken to prevent the data from being sent to unauthorized people. Patients will only be identified via their individual patient numbers throughout the entire documentation and evaluation phase, and any identifiable data will not be used.

For the personal activation of the app for each study participant, Nextlabel OHG will receive the participant’s e-mail address. To ensure the protection of all e-mail addresses, a contract has been signed between the Sponsor (University Medical Center Schleswig-Holstein) and Nextlabel OHG. The contract includes an approved data protection concept. Nextlabel will not have access to any patient data that are not pseudonymized. Amendments to the study protocol may only be implemented if approved by the responsible ethics committees. Only the coordinating principal investigator may carry out such changes. However, all coinvestigators will contact the coordinating principal investigator if modifications are deemed necessary. If any changes are made to the study protocol, all investigators will be informed after ethics committee approval, and the notice must be confirmed. 

### Data Management and Monitoring

All data relating to patients will be recorded pseudonymously. Each patient will be identifiable only by their unique patient number, date of birth, and sex. A patient identification list will only be kept in the relevant trial centers and will not be forwarded to the sponsor. Data collection will be done using the study-specific data documentation forms that must be filled in using a ballpoint pen. Fountain pens or pencils will not be used. If corrections need to be made, we will cross the error out once with a straight line, enter the correct information next to it, and note the date and/or reason for correction. Comments will be made if data fields cannot be filled in because of missing information. The forms will be filled in as soon as possible and submitted to the checker for review, signed, dated, and forwarded to the study management team.

The original versions of all key trial documents, including the documentation sheets, will be kept at the trial headquarters (ie, the sponsor responsible for the trial) for a minimum of 10 years after the final report.

The principal investigator/head of the trial center will keep all administrative documents (ie, written correspondence with the ethics committee, regulatory authorities, trial management, and trial headquarters), the patient identification list, the signed informed consent forms, copies of the documentation sheets, and general trial documentation (ie, protocol and amendments) for the aforementioned time period. Original patient data will also be kept for the length of time stipulated for the trial centers but not for less than 10 years.

Clinical on-site monitoring at the German sites will be conducted according to good clinical practices and written standard operating procedures to ensure the patients’ rights and safety are upheld, along with the reliability of the trial results. For initiation, trial sites will be visited onsite by a clinical research associate. During the trial, sites will be visited at regular intervals depending on the recruitment rate and data quality. Informed consent and defined key data will be checked for all patients. Medical files will be screened for adverse and serious adverse events. Patients’ questionnaires will be checked. All trial-specific monitoring activities will be defined before starting the trial and documented in writing (monitoring manual). The sites in other countries will be monitored according to the corresponding national regulations in their own responsibility. No regular audits are planned. However, to ensure the correct execution of the study, audits may be conducted if necessary. Because this study is not linked to the German Medicinal Products Act, no inspections of higher federal authorities are scheduled. A data monitoring committee is not required since patients in this trial will receive standard treatment.

## Results

This phase 2 trial investigates the number of radiation fractions with bladder volumes <200 ml in a prospective cohort of patients using a reminder app. In addition, this study evaluates whether the use of the app leads to a significant reduction of the proportion of fractions with bladder volumes <200 ml when compared to a historical control group without app support. The reminder app was developed by the professional company Nextlabel OHG from Lübeck, Germany. It reminds the patients to drink 300 ml of water 45 minutes prior to each radiation session. The time to be reminded can be set by the patients for each radiation session to consider potential changes in schedule (eg, due to maintenance of the linear accelerator). The patients must confirm their water intake and receive a puzzle piece as a reward. Prior to its use in the REFILL-PAC (Reminder App to Optimize Bladder Filling During Radiotherapy for Patients With Prostate Cancer) trial, the app was tested by 30 healthy volunteers to assess its functionality and practicability and to identify and solve relevant issues prior to the start of the trial. Recruitment for this trial will start in March 2025 and is planned to be completed in October 2026. Termination of the entire study (28 patients) is scheduled for December 2026. The expected results will be available at the beginning of 2027. The REFILL-PAC trial did not receive any specific funding. It is part of the Interreg Deutschland-Danmark project Health Advancing Technologies for the Elderly (HeAT) funded by the European Regional Development Fund (01-1-23 2).

## Discussion

### Expected Results

It is expected that the use of the reminder app will reduce the mean number of radiation fractions with bladder volumes <200 ml by approximately 30% from 26.7 (of 35 fractions) found in the historical control group to 18.7 fractions in patients with a bladder volume <200 ml at CT simulation [8]. A sample size of 25 patients is required for the phase 2 cohort to achieve a power of 90% for statistical significance and a 5% significance level.

### Study Limitations

Although appropriate matching will be conducted based on 7 patient- and tumor-related characteristics, the risk of a hidden selection bias cannot be entirely excluded due to the retrospective nature of the data obtained from the control group. Moreover, the fact that patients in the phase 2 cohort must possess a smartphone and be able to use the app may lead to a selection bias. These limitations must be considered when the results of the comparative part of this study will be available and distributed.

### Comparison With Prior Work

Radiotherapy for prostate cancer may be associated with significant urinary toxicity such as cystitis, particularly if the volume of the urinary bladder is <200 ml at the time of irradiation. Findings from 3 previous studies highlight the importance of patients drinking a sufficient amount of water each day before irradiation to achieve a bladder volume of <180 ml or, preferably, ≥200 ml [3-5]. To ensure appropriate bladder filling, protocols asking the patients to drink water before CT simulation and/or each radiation fraction have been investigated [5,6,8-17]. In the corresponding studies, the amount of water ranged between 200 and 600 ml, with a waiting period of 30 to 60 minutes before CT simulation or the radiation. However, bladder volumes during the radiotherapy course varied considerably, and adherence to the drinking protocols was suboptimal. This raises the question of whether an app reminding patients before each radiation session of the required water intake could improve adherence to the prescribed drinking protocol.

### Dissemination Plan

The coordinating principal investigator will work toward a comprehensive internal and external dissemination of the study results. The coordinating principal investigator, biostatisticians, and staff members of the center will prepare a report regardless of whether the study concludes as planned or is terminated early.

The scientific results will be published in an international, peer-reviewed journal. Additionally, the results will be presented at meetings and symposia. Reports and publications related to the study must be coordinated with the statistician to avoid misinterpretations. Conclusions must be statistically validated and approved by the statistician. The study acronym REFILL-PAC will be used in all publications.

### Conclusion

This phase 2 trial is the first study that investigates the impact of a reminder app on the number of radiation fractions with bladder volumes less than 200 ml in patients treated with normo-fractionated radiotherapy alone for localized prostate cancer. The hypothesis is that using the app will significantly reduce the number of fractions below 200 ml compared to a historical control group of patients who underwent normo-fractionated radiotherapy without app support. If the reminder app proves effective, it may contribute to a decrease in urinary toxicity.

## Appendix

Multimedia Appendix 1 SPIRIT (Standard Protocol Items: Recommendations for Interventional Trials) checklist.

## Abbreviations

CBCT: cone beam computed tomography
CT: computed tomography
CTCAE: Common Terminology Criteria for Adverse Events
EBRT: external beam radiotherapy
HeAT: Health Advancing Technologies for the Elderly
REFILL-PAC: Reminder App to Optimize Bladder Filling During Radiotherapy for Patients With Prostate Cancer
VMAT: volumetric-modulated arc therapy

## Data Availability

Further information regarding the REFILL-PAC (Reminder App to Optimize Bladder Filling During Radiotherapy for Patients With Prostate Cancer) trial is available at Clinicaltrials.gov (NCT06653751).

## References

[1] Siegel RL, Giaquinto AN, Jemal A (2024). Cancer statistics, 2024. CA Cancer J Clin.

[2] Leitlinienprogramm Onkologie.

[3] Pisani C, Galla A, Loi G, Beldì D, Krengli M (2022). Urinary toxicity in patients treated with radical EBRT for prostate cancer: Analysis of predictive factors in an historical series. Bull Cancer.

[4] Pinkawa M, Fischedick K, Asadpour B, Gagel B, Piroth MD, Eble MJ (2006). Low-grade toxicity after conformal radiation therapy for prostate cancer--impact of bladder volume. Int J Radiat Oncol Biol Phys.

[5] Grün A, Kawgan-Kagan M, Kaul D, Badakhshi H, Stromberger C, Budach V, Böhmer D (2019). Impact of bladder volume on acute genitourinary toxicity in intensity modulated radiotherapy for localized and locally advanced prostate cancer. Strahlenther Onkol.

[6] Pang EPP, Knight K, Hussain A, Fan Q, Baird M, Tan SXF, Mui W, Leung RW, Seah IKL, Master Z, Tuan JKL (2018). Reduction of intra-fraction prostate motion - Determining optimal bladder volume and filling for prostate radiotherapy using daily 4D TPUS and CBCT. Tech Innov Patient Support Radiat Oncol.

[7] Smith GA, Dunlop A, Barnes H, Herbert T, Lawes R, Mohajer J, Tree AC, McNair HA (2022). Bladder filling in patients undergoing prostate radiotherapy on a MR-linac: The dosimetric impact. Tech Innov Patient Support Radiat Oncol.

[8] Rades D, Cremers F, Ziemann C, Splettstösser L, Narvaez-Wolf CA, Al-Salool A, VON Staden M, Küter J, Janssen S, Timke C (2024). Changes in bladder volume during radiotherapy for prostate cancer. Anticancer Res.

[9] Chen Z, Yang Z, Wang J, Hu W (2016). Dosimetric impact of different bladder and rectum filling during prostate cancer radiotherapy. Radiat Oncol.

[10] Ingrosso G, Miceli R, Ponti E, Lancia A, di Cristino D, de Pasquale F, Bove P, Santoni R (2019). Interfraction prostate displacement during image-guided radiotherapy using intraprostatic fiducial markers and a cone-beam computed tomography system: A volumetric off-line analysis in relation to the variations of rectal and bladder volumes. J Cancer Res Ther.

[11] Chetiyawardana G, Hoskin PJ, Tsang YM (2020). The implementation of an empty bladder filling protocol for localised prostate volumetric modulated arctherapy (VMAT): early results of a single institution service evaluation. Br J Radiol.

[12] O'Doherty UM, McNair HA, Norman AR, Miles E, Hooper S, Davies M, Lincoln N, Balyckyi J, Childs P, Dearnaley DP, Huddart RA (2006). Variability of bladder filling in patients receiving radical radiotherapy to the prostate. Radiother Oncol.

[13] Chauhan K, Ebner DK, Tzou K, Ryan K, May J, Kaleem T, Miller D, Stross W, Malouff TD, Landy R, Strong G, Herchko S, Serago C, Trifiletti DM, Miller RC, Buskirk S, Waddle MR (2023). Assessment of bladder filling during prostate cancer radiation therapy with ultrasound and cone-beam CT. Front Oncol.

[14] Nathoo D, Loblaw A, Davidson M, Musunuru HB, Khojaste A, Ravi A (2018). A feasibility study on the role of ultrasound imaging of bladder volume as a method to improve concordance of bladder filling status on treatment with simulation. J Med Imaging Radiat Sci.

[15] Holden L, Stanford J, D'Alimonte L, Kiss A, Loblaw A (2014). Timing variability of bladder volumes in men receiving radiotherapy to the prostate. J Med Imaging Radiat Sci.

[16] Mullaney LM, O'Shea E, Dunne MT, Finn MA, Thirion PG, Cleary LA, McGarry M, O'Neill L, Armstrong JG (2014). A randomized trial comparing bladder volume consistency during fractionated prostate radiation therapy. Pract Radiat Oncol.

[17] Shah M, Agarwal S, Agarwal R, Subramanian B, Gupta S, De S, Mishra S (2021). Observational study of cone beam computed tomography based interfractional urinary bladder filling variation during image guided radiation therapy in pelvic malignancies. J Cancer Res Ther.

[18] Rades D, Narvaez CA, Doemer C, Janssen S, Olbrich D, Tvilsted S, Conde-Moreno AJ, Cacicedo J (2020). Radiotherapy-related skin toxicity (RAREST-02): A randomized trial testing the effect of a mobile application reminding head-and-neck cancer patients to perform skin care (reminder app) on radiation dermatitis. Trials.

[19] Rades D, Zwaan I, Cacicedo J, Bruchhage KL, Hakim SG, Olbrich D, Schild SE, Tvilsted S, Janssen S (2022). Impact of a mobile application (reminder app) on acute toxicity during radiotherapy of head-and-neck cancer - results of a randomized phase III trial (RAREST-02). BMC Cancer.

[20] Common Terminology Criteria for Adverse Events (CTCAE) version 5.0.

[21] Noether GE (1987). Sample size determination for some common nonparametric tests. J Am Stat Assoc.

